# A Rare Case of Melioidosis Breast Abscess in Far North Queensland

**DOI:** 10.7759/cureus.99073

**Published:** 2025-12-12

**Authors:** William Swee Keong Khoo, Eva Zhi Yee Teo, Alec Winder, Carly Hughes

**Affiliations:** 1 General Surgery, Townsville Hospital, Townsville, AUS; 2 Breast Surgery, Townsville Hospital, Townsville, AUS; 3 Infectious Diseases, Townsville Hospital, Townsville, AUS

**Keywords:** breast abscess, burkholderia pseudomallei, eradication of melioidosis, melioidosis, non-lactational breast abscess

## Abstract

Melioidosis is an uncommon bacterial infection caused by the organism *Burkholderia pseudomallei* and manifests in various presentations such as pneumonia, intra-abdominal infections, skin infections, and, in severe cases, sepsis. In Australia, melioidosis is mostly found in Far North Queensland. We describe a rare case of breast abscess due to *Burkholderia pseudomallei* in an immunocompromised patient in Far North Queensland, Australia. While source control by surgical drainage remains an important initial management, proper selection and duration of antimicrobial therapy and management of contributing risk factors play a key role in ensuring such patients do not present with recurrent infections, which may lead to significant morbidity.

## Introduction

Melioidosis is a rare tropical infection caused by the Gram-negative bacterial organism *Burkholderia pseudomallei*, which often resides in soil, water, and plants [1,2]. During January 1998-December 2019, the annual incidence of melioidosis in Far North Queensland, Queensland, Australia, more than doubled [3]. Patients with melioidosis have varying presentations, including pneumonia, skin infections, and intra-abdominal organ abscesses, all of which can lead to septicemia [4,5]. We present a 67-year-old female with a breast abscess secondary to *B. pseudomallei*. Her treatment comprised of surgical drainage for source control, antimicrobial therapy, and optimisation of contributing risk factors and comorbidities. This case highlights the multidisciplinary and holistic approach required in the management of melioidosis.

## Case presentation

A 67-year-old female presented to her local general practitioner for an enlarging, painful swelling with associated erythema over her left breast, clinically consistent with a non-lactational left breast abscess. The patient has a background history of poorly controlled type 2 diabetes mellitus (T2DM), psoriatic arthritis on weekly methotrexate, and is an active heavy smoker of 20 pack-years. She completed a two-week course of oral flucloxacillin with minimal improvement.

**Figure 1:**
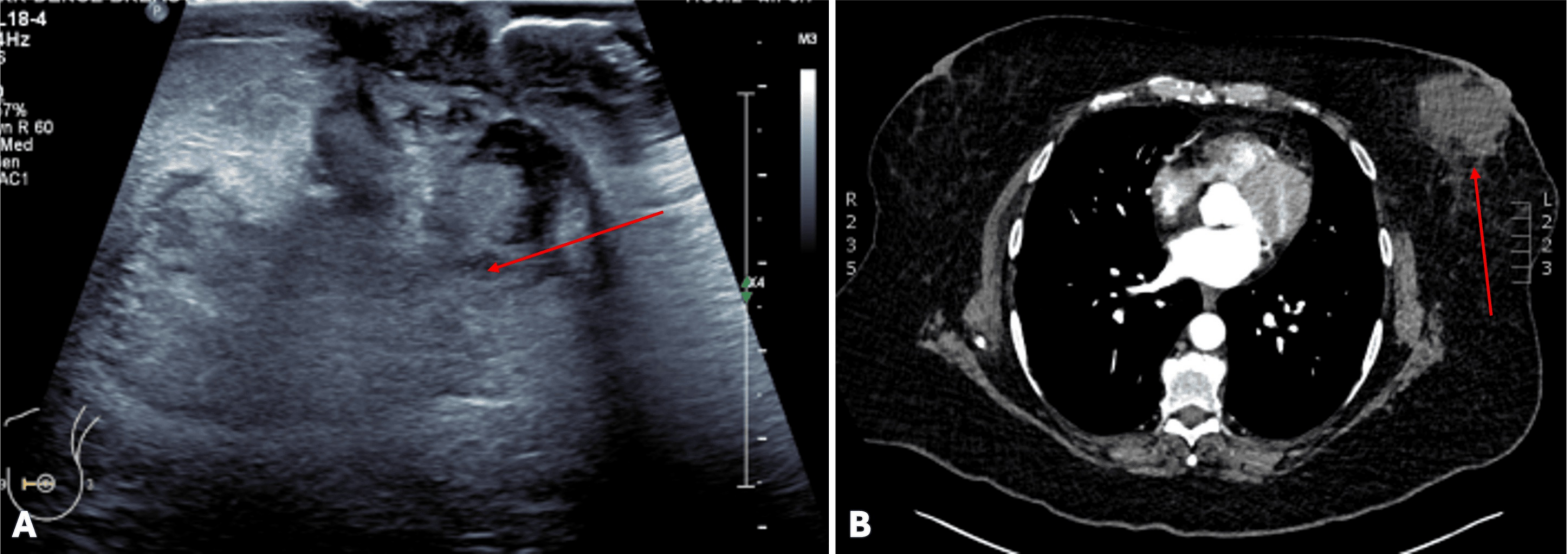
Ultrasound of the left breast and CT chest demonstrating left breast heterogenous collection consistent with a breast abscess (A: ultrasound image, B: CT chest axial view) CT: computed tomography

Given the worsening symptoms, a breast ultrasound was organised in the community, which demonstrated a large 69 x 36 x 75 mm heterogeneous collection at 9 o'clock, medial to the nipple (Figure 1A). An ultrasound-guided fluid culture was subsequently organised, which cultured *B. pseudomallei*, the causative agent of melioidosis.

She was subsequently referred to the emergency department for further management, where she was seen by the general surgical team. On admission, she was systemically well and haemodynamically stable. A physical exam revealed a tender non-fluctuant 8cm retroareolar breast swelling without features of skin necrosis, changes to the nipple, or axillary lymphadenopathy. Her initial haematological investigations revealed a normal white cell count (WCC) with an elevated Haemoglobin A1C level, indicating poor glycaemic control (Table 1). Blood cultures and a computed tomography (CT scan) of her chest, abdomen, and pelvis were organised to exclude other manifestations of melioidosis - neither of which had significant findings (Figure 1B).

**Table 1:** Patient's haematological investigations demonstrating a normal WCC and elevated HbA1C levels WCC: white cell count; HbA1c: hemoglobin A1C

Haematological investigations	Reference range	Patient’s results
White cell count (WCC)	4.0 to 11.0 10^9^/L	10.1
Haemoglobin A1C (HbA1C)	<6.5%	11.6%

The patient was commenced on intravenous meropenem 1 g eight-hourly as advised by the infectious disease physicians. This was subsequently changed to ceftazidime 2 g six-hourly after 24 hours. Given the multiloculated collection and an unsuccessful attempt at needle aspiration, surgical drainage of the left breast abscess in the operating theatre was done. Intraoperative findings revealed a large haemopurulent fluid cavity with pus culture demonstrating growth of *B. pseudomallei*. The patient was subsequently discharged home with a plan for intravenous antibiotics in the community via a peripherally inserted central catheter (PICC) to complete a four-week course of intravenous ceftazidime. Her methotrexate was also withheld for this duration.

Unfortunately, her home-based IV therapy was complicated by repeated pump errors and multiple issues with suspected PICC line occlusion. Additionally, the patient had continued smoking. The patient was subsequently re-admitted into the hospital two weeks later with a recurrence of the left breast abscess with subjective fevers. A repeat ultrasound of the breast revealed a residual 57 x 17 x 25 mm loculated collection. A combined decision was made between the surgical and infectious disease team for a second incision and drainage of the breast abscess for source control. Intraoperative cultures once again grew *B. pseudomallei*. The patient was discharged after adequate glycaemic control had been established. Upon discharge, the patient was transitioned to 3 months of oral trimethoprim-sulfamethoxazole (160/800 mg) two tablets twice daily for the eradication phase of treatment, with folic acid 5 mg daily to mitigate cytopenia risk. The patient's methotrexate was recommenced upon discharge.

The patient was subsequently followed up in both the general surgery and infectious disease clinic with serial ultrasounds demonstrating gradual resolution of the collection. A recent review upon completion of eradication therapy demonstrated a healed left breast periareolar incision with no further concerns for an abscess or infection.

## Discussion

Melioidosis is a rare tropical infection caused by the Gram-negative bacterial organism *B. pseudomallei*, which often resides in soil, water, and plants [1,2]. Melioidosis has been documented to be endemic in various parts of East Asia and Northern Australia [1,2]. During January 1998-December 2019, the annual incidence of melioidosis in Far North Queensland, Australia, more than doubled [3]. Melioidosis is strongly seasonal, with most cases reported during the summer wet season when moist soil provides the optimal conditions for *B. pseudomallei *[2,3]. Patients with melioidosis have varying presentations, including pneumonia, skin infections, and intra-abdominal organ abscesses - all of which can lead to septicemia [4,5]. Interestingly, while pneumonia remains the most common presentation of melioidosis, Australia has demonstrated a higher incidence of genitourinary infections and prostatic abscesses, whereas parotitis appears to be more prevalent in Thailand [6].

Risk factors include diabetes mellitus, hazardous alcohol use, chronic lung disease, chronic renal disease, and immunosuppression, with 20% of cases having no identifiable risk factor [7,8]. Diagnosis of *B. pseudomallei* requires isolation of the organism from the culture [2,7,9]. To date, serological diagnosis remains a challenge due to the low sensitivity in early infections [9].

The medical treatment of melioidosis can be categorised into two distinct phases: the intensive phase and the eradication phase. In the intensive phase, prompt IV antibiotics include a two-eight-week course of intravenous ceftazidime or meropenem with or without trimethoprim-sulfamethoxazole once the diagnosis is established [2,7]. This is followed by the eradication phase, which involves oral trimethoprim-sulfamethoxazole with a duration described between three and six months [2,7]. In addition, surgical drainage is recommended for source control of large abscesses. Despite considerable research and multiple vaccine development initiatives, a definitive vaccine against *B. pseudomallei* has not been achieved to date [10].

We describe a rare case of breast abscess due to *B. pseudomallei* in an immunocompromised patient in Far North Queensland, Australia. While the initial management of surgical drainage and appropriate antibiotics was administered, our patient’s poor glycaemic control, along with her other risk factors of being an active heavy smoker and being on methotrexate, resulted in a recurrence of her breast abscess.

## Conclusions

Breast abscess due to *B. pseudomallei* remains a rare and challenging presentation of melioidosis. This case highlights the importance of having a high index of suspicion for melioidosis when treating patients with breast abscesses and risk factors for melioidosis in endemic areas. While source control by surgical drainage remains an important initial management, proper selection and duration of antimicrobial therapy and management of contributing risk factors play a key role in ensuring such patients do not present with recurrent infections, which may lead to significant morbidity.
